# The Psoric Theory

**Published:** 1883-10

**Authors:** A. McNeil

**Affiliations:** Jeffersonville, Indiana


### THE PSORIC THEORY.

A. McNeil, M. D., Jeffersonville, Indiana.

None of the opinions of Hahnemann have been more bitterly assailed than the psoric theory. Arguments have been overlooked, but ridicule, misrepresentation, and unmitigated falsehoods have been lavished on it by not only allopaths, but by those calling themselves homœopaths. But one by one the old sage's opinions, after running the gauntlet of his antagonists, are adopted by them. So now an allopathic authority of illustrious name expresses views that by changing psora to diathesis might almost have been written by him. I will translate the lecture as being not only an indorsement of Hahnemann, but as being the best differential diagnosis and description of the two most important skin diseases I have ever read. Professor Guibert, in the Hôpital St. Louis, says of

### Eczema and Psoriasis:

"Gentlemen:—The traveler after his day's journey looks back over the road and with this view embraces the whole and its peculiarities. So we have traveled a long ways together and have learned what are really the dermatoses, and I have taught you that in the majority of cases *skin diseases are only the actual transfer of a multitude of inner affections* (from the mildest to the most severe) *to the skin.* Considered in this point of view, they throw light on diagnosis and pathology.

"We have studied the different anatomical disturbances which constitute diseases of the skin. You saw how that these disturbances by their changes form the different kinds of dermatoses and thereby serve to differentiate the one from the other in their individuality and distinctness.

"After this fundament consideration we have taken up the study of the individual skin diseases and began with eczema and psoriasis. The history of these affections has led to numerous details and brought forward many descriptions of the different pathological data. Let us now look back, like the traveler, and collect in our memory the different observations. Let us place together the pictures of eczema and psoriasis and consider them seperately and together. Remarkable agreements, but greater and more striking differences, will be seen.

"Eczema and psoriasis are of all skin diseases the most frequent. They are more important than all other skin diseases, not only on account of their frequency, but also because on account of their obstinacy and their tendency to extend; the functional disturbances they cause; the disfigurations to which they give rise; the length of their continuance; their tenacity; their tendency to relapses, and finally, on account of the possible frightful complications they may lead to. They are both the most general, most close related, and most clearly determined expression of that diathesis which cannot be denied, which is called herpism. (Herpetismus.) Both are hereditary, but not contagious; both belong to that great class of the excreting affections of the skin: but here their points of resemblance cease, and we meet only essential differences henceforth.

"Eczema and psoriasis are excreting diseases, but eczema is the type of *moist* excretions. The characteristic secretion begins under the epidermis, which it raises in the form of vesicles. If these are bursted, the secretion exudes on the ulcerated skin. The psoriasis, on the other hand, is the type of the *dry* secreting affections of the skin. With it there is no moisture; all is dry; the secretion is purely epidermical; it is simply the altered epidermis. That is all.

"The eczema is an inflammation. It has all the signs and characters of an inflammatory disease—congestion, redness, tension, swelling, increased temperature of the skin. The inflammation manifests itself in the moist, sticky secretions, the principal symptom. This secretion may be so profuse that it becomes a real catarrh of the skin. The inflammatory catarrh manifests itself, moreover, by subjective phenomena, *i. e.*, by disturbances in the health, morbid attacks, as feeling of tension, heat, itching, and burning. Indeed, the eczema owes its name to this feeling of burning, for it comes from the Greek word 'eksem,' I burn.

"In psoriasis all this is different. As soon as we leave eczema to turn to psoriasis we leave, as it were, the more tropical climate and go to the icy fields of the North. The eczema is the living tetter—the moist, hot tetter; psoriasis is the dry, dead tetter; its physiogony always remains without change. It remains immovable in the same stage. We have a petrified, parchment-like, mummified, dry, unsecreting skin, which no sweat moistens, which the sebacious glands no longer lubricate, which consequently has lost its softness, pliancy, elasticity, and life. About the joints and the natural openings it is unsuited to the movableness of those places and tears like an unyielding, indolent membrane; it is only a shell (carapace), a sort of scaly coat of mail without feeling, which you may scratch, wear off, or destroy without causing the slightest pain.

"The sites of eczema and psoriasis are different. The former is an inflammatory affection with most copious secretions; so it requires a warm place which is moist and stretched and relaxed much and provided with a rich vascular network, such as is the case in the neighborhood of the sexual organs and the axillæ. Psoriasis, on the other hand, requires only much epidermis, and therefore develops on those parts which are richly provided with it. If you will consider the different sites, look at the lower extremities. In the bend of the knee you meet eczema; *on* the knee, psoriasis. On the upper, you perceive in the bend of the elbow eczema; *on* the elbow, psoriasis. However, there are certain places in which both flourish, just as plants which prefer moist soil yet can grow on a more or less dry one. So eczema and psoriasis may be met with *in all* parts of the body. But the character of each is more or less modified, exactly as in the above-mentioned plants when they are transferred to a less congenial *habitat*.

"Eczema and psoriasis differ in the character of their accompanying diseases. The complications of eczema are of an inflammatory character, as itself is inflammatory in its nature. Let this inflammation be considerable or not, yet it always shows its eczematous relation. It may extend to the entire thickness of the skin, into the cellular tissue, the subcutaneous tissue, the lymphatic glands; it may even take on an erysipelous or a phlegmonous character, or become a lymphangioitis with its ramifications and its rose-colored and bead-like cords. *These complications are at times deep-reaching, even extending to the viscera; they may attack the great apparatus of the system, the nerve centres, the digestive and respiratory organs. Also meningitis, acute inflammation of the brain, bronchial and gastro-intestinal catarrhs arise therefrom.* But these complications are always of an acute, intense character, corresponding to the acuteness and intensity of the eczema, to which they owe their origin.

"Psoriasis, on the contrary, with its *chronic* type, has only complications of chronic character. Those of the lungs are mostly chronic catarrh not seldom ending in tuberculosis; those of the digestive organs, dyspepsia, different varieties of cancer, carcinoma of the intestines, and still more frequently of the stomach.

"Eczema and psoriasis are also different in their course and development. The former comes with an acute beginning, the latter with a chronic one. It is a torpid type; it has a slow course, or rather it does not move forward at all, it remains what it is. It is to-day what it was yesterday, and to-morrow it will still be the same.

"In its fourth stage eczema is scaly, like psoriasis, but these eczematous scales are essentially different. They are delicate, laminated, are not transparent, and contain in the layer of epidermis some moisture; they loosen in larger or smaller flakes and are very easily removed from the cuticle underneath. The psoriasis scales are dense, and so closely interwoven in its parts that it can only be loosened in the form of dust. There is never the slightest trace of moisture.

"And yet, gentlemen, these diseases so contrary, these two opposite poles of dermatology, may in some cases coalesce and unite in order to make a bastard form which partakes of the qualities of both without being either one or the other. Just as there is an eczema lichenoides, which is a coalescence of eczema and lichen, so, notwithstanding what the learned Hardy said, an eczematous psoriasis consisting of eczema and psoriasis may exist. You will perceive, for example, the strong, thick scales of psoriasis, but on their inner side there is a crusty element; they are loosened from a somewhat moist skin. It has something of the nature of eczema; it is really an eczematous psoriasis.

"Eczema and psoriasis are two general affections, which by preference are situated on the surface; they may give rise to most violent complications, from a simple catarrh of the bronchia to meningitis, tuberculous pneumonia, and even to gastric and intestinal cancer."

